# Effects of low-fat diet and aging on metabolic profiles of *Creb3l4* knockout mice

**DOI:** 10.1038/nutd.2015.29

**Published:** 2015-08-24

**Authors:** T-H Kim, J-M Park, S-H Jo, M-Y Kim, H Nojima, Y-H Ahn

**Affiliations:** 1 Department of Biochemistry and Molecular Biology, Yonsei University College of Medicine, Seoul, Republic of Korea; 2 Department of Molecular Genetics, Research Institute for Microbial Diseases, Osaka University, Osaka, Japan; 3 Brain Korea 21 PLUS Project for Medical Science, Yonsei University College of Medicine, Seoul, Republic of Korea

## Abstract

**Background/Objectives:**

Increased adipose tissue mass closely associates with the development of insulin resistance and type 2 diabetes mellitus. Previously, we reported that CREB3L4 expressed in adipose tissue negatively regulates adipogenesis, and *Creb3l4* knockout mice fed a high-fat diet for 16 weeks showed fat cell hyperplasia, with improved glucose tolerance and insulin sensitivity. These mice did not show significant weight gain and fat mass. Because fat diet or aging is known to be associated with the development of obesity, we examined the effects of *Creb3l4* gene subjected to low-fat diet (LFD) or aging process on body composition and obesity risk.

**Subjects/Methods:**

We fed *Creb3l4* knockout mice a low-fat diet for 16 weeks (LFD group) or chow diet for over 1 year (aged group) and observed various metabolic parameters in the LFD-fed and aged *Creb3l4* knockout mice.

**Results:**

LFD-fed and aged *Creb3l4* knockout mice showed significant weight gain and adiposity, impaired glucose tolerance and decreased insulin sensitivity, compared with wild-type mice.

**Conclusions:**

*Creb3l4* has a critical role in metabolic phenotypes and a better understanding of its function may provide improved insight into the etiology of diabetes and other metabolic disorders.

## Introduction

Western societies have adopted a lifestyle with low physical activity and overconsumption of energy-rich food,^1^ resulting in a substantial increase in the incidence of obesity accompanied by impaired glucose and lipid metabolism.^1, 2, 3^ In general, it is accepted that expansion of adipose tissue and lipid accumulation in insulin-sensitive tissues (for example, skeletal muscle and liver) closely relates to the development of metabolic diseases.^4^ In particular, increased adipose tissue mass closely associates with the development of insulin resistance and type 2 diabetes mellitus.^5^ In addition, increased adiposity is a hallmark of aging and a source of chronic inflammation, which further accelerates the aging process. This vicious cycle of aging, visceral fat accumulation and inflammation often leads to metabolic dysfunction.^6, 7^ Thus understanding the molecular mechanism(s) of adipose tissue expansion in the context of transcriptional regulation could be pivotal to combat metabolic syndromes, including obesity and type 2 diabetes mellitus.

The endoplasmic reticulum and Golgi transmembrane protein CREB3L4 (cAMP responsive element binding protein 3-like 4) is mainly expressed in human prostate tissue and mouse testis.^8, 9^ Specifically, CREB3L4a, also known as Atce1/Tisp40a, is the major form of CREB3L4 expressed in mouse testis^8^ and is highly homologous with mouse CREB3 (identity 62%, similarity 72%).^10^ Deletion of the transmembrane domain of CREB3L4 results in nuclear accumulation regulated by endoplasmic reticulum stress.^11^ Moreover, whole-body knockout of *Creb3l4* (*Creb3l4* KO) results in abnormal epididymal sperm nuclei.^12^ CREB3L4 is also known to have an important role in the development of prostate cancer and is more highly expressed in cancerous than in non-cancerous prostate cells.^13^ However, the functional role of CREB3L4 in insulin-sensitive tissues such as adipose tissue, skeletal muscle or liver is not well known.

Recently, we reported that CREB3L4 could regulate adipocyte differentiation.^14^ Upon CREB3L4 downregulation, adipocyte differentiation occurs even when cultured with a minimal hormonal inducer.^14^ In addition, we showed that *Creb3l4* KO mice fed a high-fat diet (HFD) did not show significant differences in body weight or fat mass, as compared with wild-type (WT) controls.^14^ However, KO mice undergo fat cell hyperplasia owing to increased adipogenesis, with improved glucose tolerance and increased insulin sensitivity.^14^

To examine whether the process of aging and low-fat diet (LFD) influences metabolic phenotypes, we explored the effects of an LFD or aging on the metabolic profiles of *Creb3l4* KO mice. Both LFD (LFD group) and aged *Creb3l4* KO mice fed chow diet (aged group) were prone to body weight gain with increased adiposity. These mice also exhibited impaired glucose tolerance and decreased insulin sensitivity, as compared with WT control mice.

## Materials and methods

### Animal experiments and KO mice

All animal experiments were approved by the Institutional Animal Care and Use Committee of Yonsei University Cmollege of Medicine (Protocol no. 2011-0199, 2011-0315, 2012-0255). All mice were housed under standardized conditions of humidity, temperature (22–24 °C) and a 12-h light/12-h dark cycle. The male mouse (11 weeks, *n*=4–5) strains *db/m*^*+*^(C57BLKS/J lar-m^+^/Lepr^db^), *db/db* (C57BLKS/J lar-Lepr^db^/Lepr^db^), lean and *ob/ob* (C57BL/6J Ham Slc-*ob/ob*) (Shizuoka laboratory, Hamamatsu, Japan) were fed a regular chow diet. The *Creb3l4* KO mouse has been described previously,^12^ and *Creb3l4* KO mouse embryos were purchased from RIKEN (The Institute of Physical and Chemical Research, Ibaraki, Japan) with permission from Dr Nojima. New born pubs of *Creb3l4* KO mice and WT littermates were randomly selected by genotyping PCR using specific primer. *Creb3l4* KO mice and their WT littermates were 10–11-week old and weighed approximately 23 g; the mice were fed a 10% LFD (D12450B, Research Diets, New Brunswick, NJ, USA) over 16 weeks. The body weight of mice was assessed every weekly. To observe an effect of aging process, the aged WT and aged *Creb3l4* KO mice (*n*=3–6) were fed a regular chow diet for up to 85 weeks.

### Glucose and insulin tolerance tests

For oral glucose tolerance tests, mice were fasted for 16 h and then orally administered glucose (20% wt/vol.) using a feeding tube (2 g kg^−1^ body weight), with blood glucose levels monitored at each time point. For insulin tolerance tests, mice were fasted for 6 h and then given an intraperitoneal injection of insulin (0.75 U kg^−1^ body weight; Humulin R, Eli Lilly, Indianapolis, IN, USA). Blood glucose levels were monitored (One TOUCH Sure Step, Life Scan, Milpitas, CA, USA) at the time points indicated in the figure legends. The areas under the curve of glucose were calculated during the course of the tests.

### Phenotypic evaluation of mice

WT male and *Creb3l4* KO male mice were fed a 10% LFD for 18 weeks. Plasma insulin and adiponectin levels were then measured using enzyme-linked immunosorbent assay kits (ALPCO Immunoassays, Salem, NH, USA). Plasma resistin was measured using MAGPIX (Luminex, Austin, TX, USA) with MILLIPLEX MAP mouse magnetic beads (Merck Millipore Corp., St Charles, MO, USA).

### Quantitative real-time PCR

Total RNA was isolated from mouse adipose tissue using TRIzol reagent (Invitrogen, Carlsbad, CA, USA) according to the manufacturer's instructions, and cDNA was generated using a reverse transcription system (ImProm-II Reverse Transcription System; Promega, Madison, WI, USA). Quantitative real-time PCR was performed using a real-time PCR system (Step One, Applied Biosystems, Foster City, CA, USA), according to the manufacturer's protocol. Changes in mRNA levels were calculated using the comparative Ct method.^15^ The relative amount of mRNA in each sample was normalized to transcript levels of the *Rplp0* (*36B4*) ribosomal protein-encoding housekeeping gene. Primers used for real-time PCR were as follows: *Creb3l4*-F, 5′-ATATCTTCTCGACGGGATCCTT-3′ *Creb3l4*-R, 5′-TCCCTACCAGGAGATGTTTC; *Cidec*-F, 5′-TCCAGGACATCTTGAAACTT-3′ *Cidec*-R, 5′-GGCTTGCAAGTATTCTTCTGT-3′ *Cd36*-F, 5′-TGCACCACATATCTACCAAA-3′ *Cd36-*R, 5′-TTGTAACCCCACAAGAGTTC-3′ *Pepck*-F, 5′-ACACACACACATGCTCACAC-3′ *Pepck*-R, 5′-ATCACCGCATAGTCTCTGAA-3′ *Fasn*-F, 5′-TTTGCTGCCGTGTCCTTCTACC-3′ *Fasn*-R, 5′-ATGTGCACAGACACCTTCCCGT-3′ *Nr1h3(Lxra)-*F, 5′-GAGAAGCTGGTGGCTGCCCA-3′ *Nr1h3(Lxra)*-R, 5′-AGCTGTAGGAAGCCAGGGAG-3′ *Cd11c*-F, 5′-ACACAGTGTGCTCCAGTATGA-3′ *Cd11c*-R, 5′-GCCCAGGGATATGTTCACAGC-3′ *Emr1(F4/80)*-F, 5′-CTTTGGCTATGGGCTTCCAGTC-3′ *Emr1(F4/80)*-R, 5′- GCAAGGAGGACAGAGTTTATCGTG-3′ *Rplp0*-F, 5′-TGGCCAATAAGGTGCCAGCTGCTG-3′ and *Rplp0*-R, 5′-CTTGTCTCCAGTCTTTATCAGCTGCAC-3′.

### Hematoxylin and eosin staining

Liver, brown adipose tissue (BAT), epididymal white adipose tissue (WAT) and subcutaneous WAT were fixed with 10% neutral-buffered formalin, embedded in paraffin and sectioned. Hematoxylin and eosin staining was then performed on these sections.

### Body composition analysis

Body compositions of WT and *Creb3l4* KO mice were determined using non-invasive quantitative magnetic resonance relaxometry on an EchoMRI-900 (Echo Medical Systems, Houston, TX, USA) at the Phenogenomic Research Center, Woo Jung BSC, Inc., Korea. Scans were then performed by placing animals in a thin-walled plastic cylinder (3-mm thick, 4.5-cm inner diameter, based on mouse body weight), which was inserted into a mouse cylindrical sensory antenna, the A100, to limit movement. All quantitative magnetic resonance measurements were made during the light phase (0700–1900 hours). The accumulation factor used was set for extra-high precision ( × 3), resulting in a scan time of approximately 2.5 min.

### Statistical analysis

Data are represented as means±s.e.ms. The LFD experiment was repeated at least three times. For aging study, experiment was performed once. All data sets were analyzed for statistical significance using nonparametric Mann–Whitney tests or Student's *T*-tests. All *P-*values <0.05 were considered significant. Statistical analyses were carried out using SPSS (IBM SPSS statistics ver. 20; IBM Corp., Armonk, NY, USA).

## Results

### *Creb3l4* KO mice fed an LFD show increased adipocyte hypertrophy and obesity

**Figure 1:**
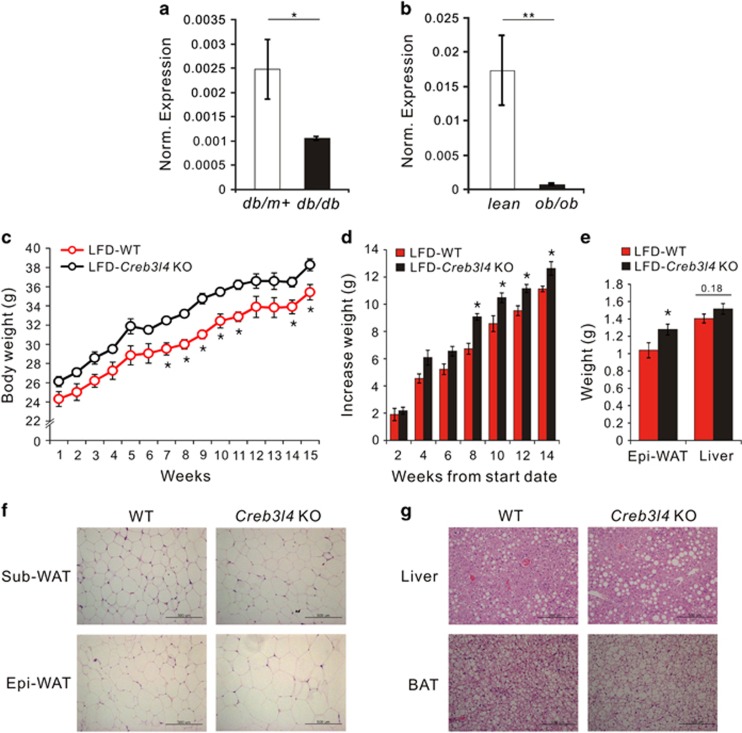
Effects of an LFD on *Creb3l4* KO mice. Real-time PCR analysis of *Creb3l4* mRNA levels in epididymal white adipose tissue (Epi-WAT) from *db/db* (**a**) and *ob/ob* (**b**) mice. The mRNA expression levels were normalized to those of *Rplp0* (*36B4*) as a control. (**c**) Body weight and (**d**) increases in body weight of WT and *Creb3l4* KO mice observed for 15 weeks on an LFD. (**e**) Weight and increased weight of Epi-WAT and liver in LFD-fed WT and *Creb3l4* KO mice. (**f**) Hematoxylin and eosin (H&E) staining of subcutaneous white adipose tissue (Sub-WAT) and Epi-WAT from WT and *Creb3l4* KO mice. (**g**) H&E stain of the liver and BAT. Scale bar, 500 μm. Values are expressed as means±s.e.ms., *n*=4–5 in panels (**a**–**d**), *n*=12–14 in panel (**e**), *P<0.05, **P<0.01.

Because *Creb3l4* is known to be expressed in WAT,^14^ we examined the mRNA levels of *Creb3l4* in WAT of *ob/ob* and *db/db* mice to test whether obesity correlated with *Creb314* gene expression. *Creb3l4* mRNA levels in WAT of these mice were lower than those in the WT controls (Figures 1a and b). The reduced *Creb3l4* expression in WAT of these mice led us to explore a cause–effect relationship between CREB3L4 and the development of adiposity.

To observe the effect(s) of CREB3L4 on whole body metabolism, we fed *Creb3l4* KO mice an LFD and observed body weight changes over 16 weeks. LFD induced a significant gain in body weight (Figures 1c and d) in *Creb3l4* KO mice that associated with increased weight of epididymal WAT, as compared with their WT littermates (Figure 1e). Although the food intake was not significantly different between WT and *Creb3l4* KO mice, the *Creb3l4* KO mice were likely to consume more food (Supplementary Figure S1a). Liver weight was not significantly different between these mice (Figure 1e). The size of subcutaneous and epididymal fat cells in *Creb3l4* KO mice were larger than those in WT mice (Figure 1f). In addition, lipid accumulation in the liver and BAT increased in *Creb3l4* KO mice (Figure 1g). These results suggest that *Creb3l4*-null mice fed an LFD incur adipocyte hypertrophy and obesity, with lipid accumulation in the liver and BAT, in addition to increased adipose tissue weight.

### *Creb3l4* KO mice fed an LFD exhibit impaired glucose tolerance and decreased insulin sensitivity

**Figure 2:**
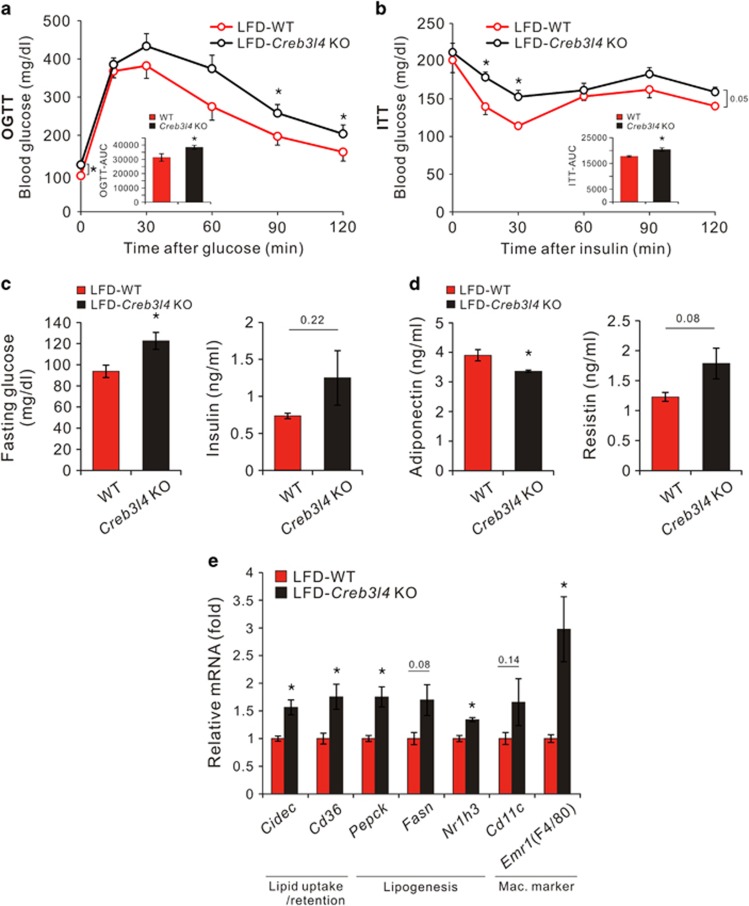
*Creb3l4* KO mice show impaired glucose tolerance and decreased insulin sensitivity. (**a**, **b**) Oral glucose tolerance test (OGTT) and insulin tolerance test (ITT) responses (0–120 min) performed after 14 and 15 weeks of LFD feeding, respectively (**c**, **d**) Fasting glucose, plasma insulin, adiponectin and resistin levels from WT or *Creb3l4* KO mice fed an LFD for 16 weeks. (**e**) Relative expression of adipogenic genes, macrophage marker genes and lipogenenic genes in epididymal white adipose tissue, normalized to the housekeeping gene *Rplp0*. Values are expressed as means±s.e.ms., *n*=6–7 in panels (**a**) and (**c**), *n*=4–5 in panels (**b**, **d** and **e**), *P<0.05 for *Creb3l4* KO versus WT mice.

Because obesity leads to insulin resistance, we assessed glucose tolerance and insulin sensitivity in WT and *Creb3l4* KO mice. *Creb3l4* KO mice fed an LFD (LFD-*Creb3l4* KO) showed impaired glucose tolerance and insulin sensitivity, as compared with WT littermate mice (Figures 2a and b).

Fasting glucose and insulin levels were also increased in LFD-*Creb3l4* KO mice, typical signs of insulin resistance (Figure 2c). However, plasma lipid profiles, including triglycerides, total cholesterol and non-esterified free fatty acid levels, were not significantly different between these groups (Supplementary Figure S1b). Because adipose tissue is known to control systemic glucose homeostasis by secreting adipokines, we measured adipokine plasma levels. The LFD-*Creb3l4* group showed decreased serum adiponectin, but increased resistin levels, compared with levels in WT mice (LFD-WT). These results suggest that LFD-*Creb3l4* mice possess an altered adipokine profile that would aggravate insulin sensitivity (Figure 2d). Moreover, the expression of the *cell death-inducing DFFA-like effector c* (*Cidec*) and *Cd36*, both related to lipid uptake/retention, was also increased in adipose tissue of LFD-*Creb3l4* KO, as were the mRNA levels of the lipogenic genes *phophoenolpyruvate carboxykinase* (*Pepck*), *fatty acid synthase* (*Fasn*) and *nuclear receptor subfamily 1, group H, member 3* (*Nr1h3, lxra*) (Figure 2e).

Obesity is known to be a state of chronic low-grade inflammation with infiltration of adipose tissue macrophages. Adipose tissue macrophages are characterized by the presence of F4/80^+^ and CD11b^+^, with subtypes M1 and M2 classified by the presence (M2) or absence (M1) of CD11c.^16^ As shown in Figure 2e, adipose tissue macrophages, particularly the M2 subtype, strongly infiltrated the epididymal fat in LFD-*Creb3l4* KO mice, as compared with LFD-WT mice. These results are consistent with data showing increased fat mass and adipocyte size (hypertrophy) in LFD-*Creb3l4* KO mice (Figures 1c and g). These findings further suggest that the WAT of *Creb3l4*-deficient mice, when fed an LFD, may undergo increased fat mass that impairs glucose tolerance and decreases insulin sensitivity. In addition, lipid accumulation in the liver and BAT may also contribute to defects in glucose tolerance and insulin sensitivity (Figure 1g).

### Aged-*Creb3l4* KO mice fed chow diet show increased adiposity with impaired glucose tolerance and decreased insulin sensitivity

**Figure 3:**
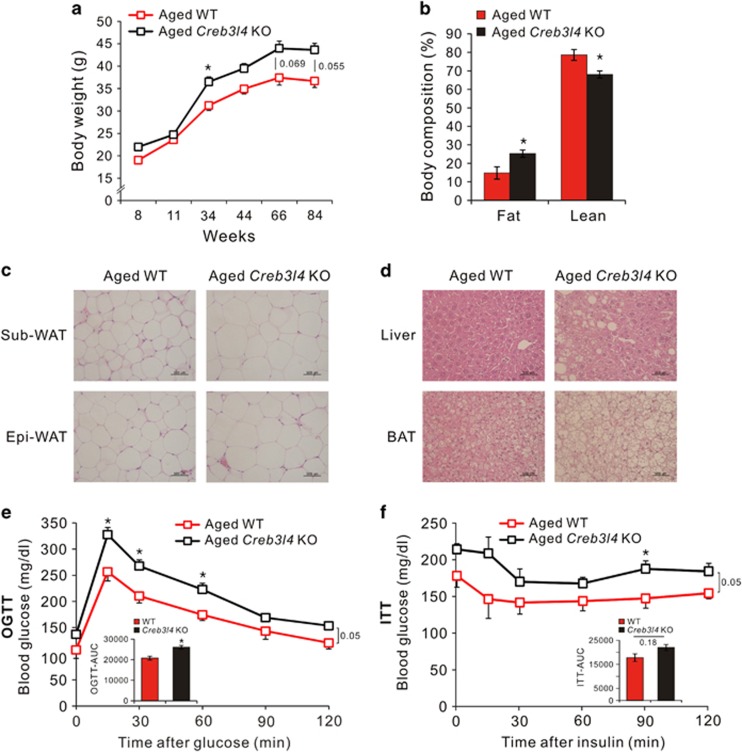
Aged-*Creb3l4* KO mice fed a chow diet show increased adiposity with impaired glucose tolerance and decreased insulin sensitivity. (**a**) Body weight changes in WT and *Creb3l4* KO mice fed a chow diet for 84 weeks. (**b**) Body composition (% body weight) analysis of WT and *Creb3l4* KO mice. (**c**) Hematoxylin and eosin (H&E) stain of subcutaneous white adipose tissue (Sub-WAT) and epididymal white adipose tissue (Epi-WAT) from aged-WT and aged-*Creb3l4* KO mice (**d**) H&E stain of the liver and BAT from aged-WT and aged*-Creb3l4* KO mice. Oral glucose tolerance test (OGTT) (**e**) and insulin tolerance test (ITT) (**f**) were performed after 90 weeks glucose intake and 92 weeks insulin adminstration, respectively. Values are expressed as means±s.e.ms., *n*=3–6 in panels (**a**, **b**, **e** and **f**). *P<0.05 for *Creb3l4* KO versus WT.

Aging is an important risk factor for metabolic disorders, including obesity, impaired glucose tolerance and type 2 diabetes.^7, 17^ To further investigate an effect of CREB3L4 on age-related adiposity, both WT and *Creb3l4* KO mice were fed a chow diet for up to 85 weeks, resulting in significantly increased body weight of the aged-*Creb3l4* KO mice, compared with the body weight of aged-WT mice (Figure 3a). The fat composition of *Creb3l4* KO mice also increased (Figure 3b), as did fat cell size, in aged-*Creb3l4* KO mice, compared with aged-WT mice (Figure 3c). Lipid accumulation in the liver and BAT also increased in aged-*Creb3l4* KO mice (Figure 3d). Aged-*Creb3l4* KO mice also showed impaired glucose tolerance and decreased insulin sensitivity, compared with their WT littermates (Figures 3e and f), a phenomenon we also observed in LFD-fed mice.

## Discussion

As accumulation of fat is known to be related to insulin resistance, obesity and metabolic syndrome, studies of associated transcription factor are critical to development of therapy. Based on previous studies which showed that inhibition of *Creb3l4* induced adipogenesis,^14^ we examined whether *Creb3l4* affects fat mass in mice fed an LFD and aged mice with chow diet. Increased fat mass, with hypertrophic WAT, was present in LFD-fed *Creb3l4* KO mice and aged-*Creb3l4* KO mice, which likewise exhibited impaired glucose tolerance and decreased insulin sensitivity, presumably caused by upregulated lipogenic genes in their epididymal WAT. Increased expression of lipogenic genes in the WAT of *Creb3l4* KO mice could contribute to accumulation of triglyceride in adipocytes. Triglyceride content was also increased in the liver and BAT of LFD-*Creb3l4* KO mice. There is no difference of energy expenditure and physical activity between WT and *Creb3l4* KO mice (Supplementary Figure S2). But the *Creb3l4* KO mice showed tendency to uptake more food (Supplementary Figure S1a), which may contribute to higher body fat contents in LFD-fed *Creb3l4* KO mice than LFD-fed WT mice.

Furthermore, because *Creb3l4* KO mice used in this study is whole-body KO, we could not rule out indirect effect of the absence of *Creb3l4* on lipid homeostasis. Detailed relationship between lipogenesis and *Creb3l4* gene needs further research. In contrast, HFD-fed *Creb3l4* KO mice showed no significant differences in body weight or fat mass, with adipocyte hyperplasia improving glucose tolerance and insulin sensitivity, as compared with WT controls.^14^ However, these findings raise the question of why do adipocyte responses in *Creb3l4* KO mice fed an LFD differ from those fed a HFD? Moreover, why do LFD-fed *Creb3l4* KO mice showed decreased insulin sensitivity (with adipocyte hypertrophy), whereas HFD-fed *Creb3l4* KO mice exhibit increased insulin sensitivity with adipocyte hyperplasia, as compared with WT mice? We speculated that the phenotype of HFD-*Creb3l4* KO mice may arise owing to increasingly generated adipocyte precursors, arising from embryonic stem cells and their self-renewal, being recruited to WAT, resulting in adipocyte hyperplasia that improves glucose tolerance. Indeed, high dietary fat content is known to expand adipose tissue mass by recruiting white adipocytes from progenitor cells.^18, 19^ In addition, HFD-induced adipose tissue expansion is triggered by hypertrophy during the first month of HFD feeding, after which adipose tissue expands by formation of a large number of new fat cells (hyperplasia),^20^ which are known to increase glucose disposal and insulin sensitivity.^21, 22, 23^ At present, it is not clear whether LFD induces formation of new fat cell. However, as it was shown that chow diet did not induce adipogenesis,^20^ we speculate that the response and role of *Creb3l4* may differ from food (diet) type in terms of adipogenesis, resulting in different body fat content, as manifested by opposite patterns of lipogenesis versus lipolysis, resulting in weight gain in the *Creb3l4* KO mice fed HFD versus LFD diets, as compared with WT.

Indeed, because *Creb3l4* is mainly expressed in the stromal vascular fraction, an adipose tissue subpopulation of various progenitor and immune cells,^14^ we could not exclude the possibility that *Creb3l4* may have a role in the formation and recruitment of adipocyte precursors to WAT, based on diet (for example, LFD or HFD) that affects adipocyte size. These observations, together with the presence of *Creb3l4* in the stromal vascular fraction in WAT, might explain another potential role of *Creb3l4* in the transition of embryonic stem cells into preadipocytes, leading to the increased numbers of adipocytes observed in HFD-*Creb3l4* KO mice. In addition, in contrast to the HFD group, the LFD regimen consisted of 35% sucrose, rather than fat. Fructose, derived from sucrose, can affect carbohydrate metabolism by changing intermediate glycolytic metabolites and gene expression patterns of metabolism-related enzymes.^24^ Moreover, high consumption of fructose causes hepatic steatosis and obesity, while also eliciting metabolic abnormalities such as insulin resistance, leptin resistance and hypertriglyceridemia.^24, 25^ Thus it is speculated that different phenotype observed in *Creb3l4* KO mice, in response to different diets, might be due to the sucrose disaccharide contained in the LFD diet.

Thus LFD and/or aged *Creb3l4* KO mice showed increased adiposity with hypertrophic WAT, which may contribute to the development of impaired glucose tolerance and decreased insulin sensitivity. Because WAT expresses more *Creb3l4* mRNA than other metabolic tissues,^14^ this metabolic phenotype may result from altered CREB3L4 action in WAT. However, in the absence of WAT-specific *Creb3l4* KO mice, we cannot rule out that *Creb3l4* deficiency in other tissues also contributes to the phenotype observed in these mice.

In summary, the aging process, or consumption of an LFD, in *Creb3l4* KO mice showed different phenotypes than those fed a HFD. Thus, understanding the molecular mechanism of differential regulation of adiposity in *Creb3l4* KO mice fed differing diet or aging may provide critical clues in combating metabolic syndromes associated with obesity.
