# Supplementary material for: Effects of low-fat diet and aging on metabolic profiles of Creb3l4 knockout mice
Source: Nutr Diabetes. 2015 Aug 24;5(8):e179–. doi: 10.1038/nutd.2015.29 (PMC4558560; doi:10.1038/nutd.2015.29)
Supplement: Supplementary Information [file nutd201529x1.docx]

Supplementary Information


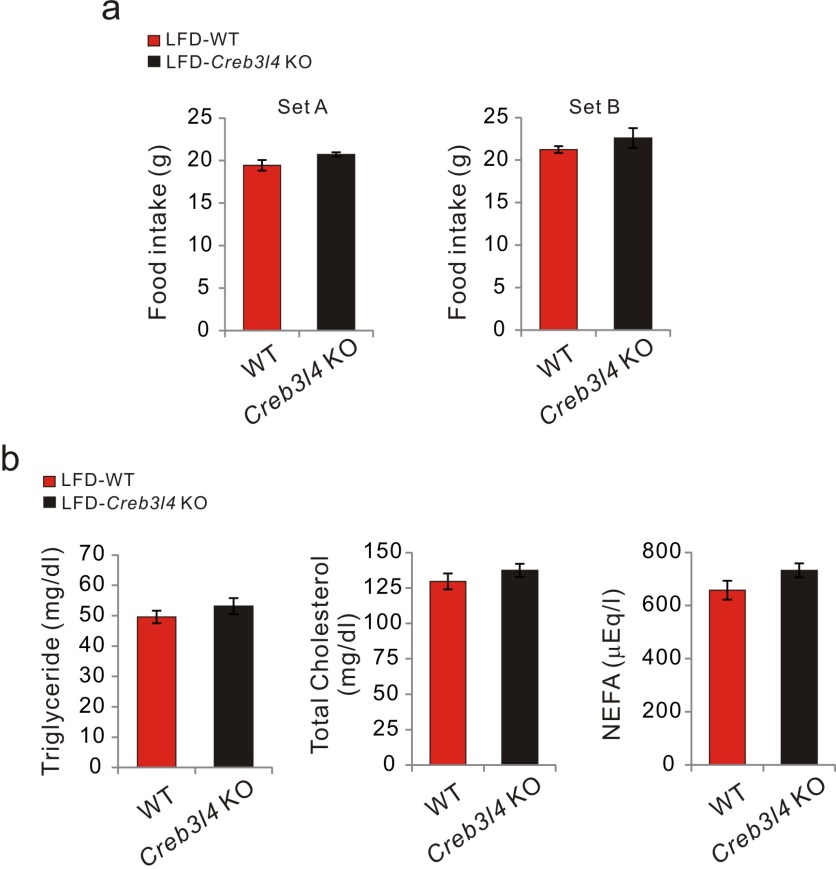


**Supplementary Figure 1.** Food intake and plasma lipid parameters of low fat diet (LFD) fed mice. (a) Food intake of wild type (WT) and *Creb3l4*KO mice were measured for 7 days, which were repeated twice (Set A, and Set B, n=4-5), respectively. (b) Serum lipid profiles, triglyceride, total cholesterol, and non-esterified fatty acid (NEFA) levels were measured by enzymatic methods using an autoanalyzer (n = 13-15). Values are expressed as mean ± SEM.


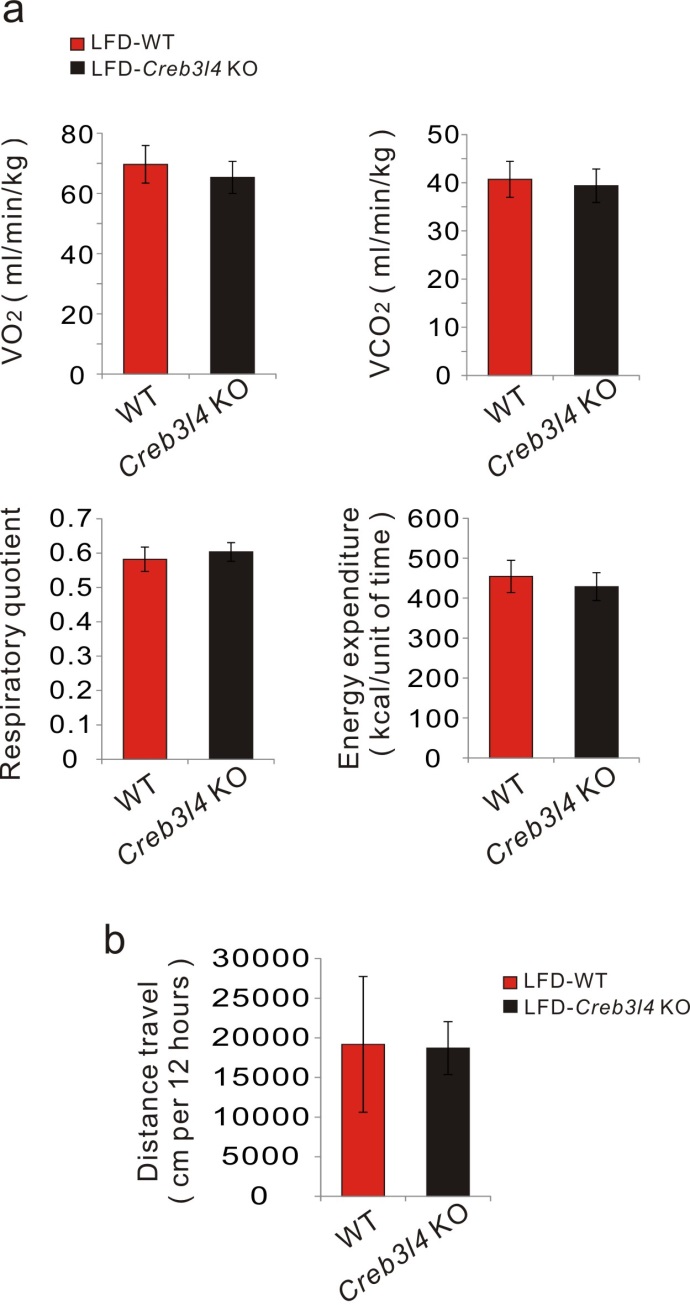


**Supplementary Figure 2.** The metabolic parameters of LFD fed mice. (a and b) The parameters were determined using the PhysioScan Metabolic System. Mice were acclimatized to cages for 48h and mice were monitored in the metabolic chamber for 12h with no access to feed and water. n = 3 per group

**Supplementary Materials and Methods**

Indirect calorimetry

The indirect calorimetry, Activity levels were determined using the PhysioScan Metabolic System (AccuScan Instruments, Inc., Columbus, OH, USA). This system utilizes zirconia and infrared sensors to monitor oxygen (O_2_) and carbon dioxide (CO_2_), respectively, inside of respiratory chambers in which individual mice were tested. All comparisons are based on animals studied simultaneously in four different chambers connected to the same O_2_ and CO_2_ sensors in an effort to minimize the effect of environmental variations and calibration on data. After a 48-h acclimation period, mice were monitored in the metabolic chambers for 12 h with no access to feed and water. Gas samples were collected and analyzed every ten minutes per animal, and the data were averaged for each hour. Output parameters include oxygen consumption (VO_2_, mL kg^-1^ min^-1^), respiratory quotient (RQ, VCO_2_ ⁄ VO_2_) and activity (Distance travel, cm).

Plasma lipid profiles

Plasma metabolites, such as cholesterol, triacylglycerol and NEFA were measured by an enzymatic method using an autoanalyser (Hitachi 7600: Hitachi Instruments, Tokyo, Japan) as per manufacturer’s instructions.
